# Translation, cross-cultural adaptation and preliminary application of the premature infant oral motor intervention in Chinese neonatal nursing practice: a pilot randomized controlled trial

**DOI:** 10.3389/fped.2026.1784065

**Published:** 2026-05-29

**Authors:** Nana Wu, Xia Chen, Jie Fu, Liwen Ding, Shuang Wu, Sushan Yuan, Hong Zhou

**Affiliations:** Neonatal Intensive Care Unit, Chengdu Women’s and Children’s Central Hospital, School of Medicine, University of Electronic Science and Technology of China, Chengdu, China

**Keywords:** cross-cultural adaptation, e-Delphi, PIOMI, premature infants, translation

## Abstract

**Background:**

Oral motor intervention plays a critical role in facilitating nutritional intake and supporting neurodevelopmental outcomes in preterm infants. The Premature Infant Oral Motor Intervention (PIOMI) is a standardized, five-minute, eight-step manual protocol designed to enhance oral-motor skills and sucking performance in very preterm infants.

**Aim:**

To translate, culturally adapt, and preliminarily validate the PIOMI for neonatal nurses in mainland China, and to pilot-test its clinical feasibility and preliminary efficacy on oral sucking performance in very-preterm infants.

**Design:**

This study employed a two-phase translation and testing approach. Phase 1 comprised Brislin's six-step translation, a two-round electronic Delphi (e-Delphi) process, and assessment of content validity and implementation consistency. Phase 2 involved a parallel-group pilot randomized controlled trial.

**Methods:**

This study cross-culturally adapted the Premature Infant Oral Motor Intervention (PIOMI) into Chinese using Brislin's framework, finalized the mainland Chinese version (CMV-PIOMI) via a two-round e-Delphi expert consensus (*n* = 21), assessed its implementation consistency (*n* = 117), and evaluated its efficacy on preterm infants' oral sucking ability through a randomized controlled trial (*n* = 76).

**Results:**

The Delphi response rate was 85.71% (*n* = 18), I-CVI ranged from 0.88 to 1.00, and S-CVI/Ave was 0.96, with six amendments incorporated. Among the 117 NICU nurses, the Pearson correlation coefficient for implementation consistency was 0.68 (*p* < 0.001), and paired CMV-PIOMI operational scores did not differ systematically (27.95 ± 0.48 vs. 27.99 ± 0.45, *p* = 0.266). In the pilot RCT (*n* = 76), baseline characteristics were comparable, and the proportion of preterm infants with normal sucking patterns was higher in the intervention group (50.0%) than in the control group (21.1%) (95% CI: 7.0% to 47.2%; *p* = 0.008).

**Conclusions:**

The Chinese PIOMI shows satisfactory content validity and acceptable implementation consistency and is feasible for delivery by bedside nurses. Preliminary evidence suggests improved oral sucking performance in very preterm infants, but larger, fully powered multicenter trials are needed to confirm these initial findings.

**Clinical Trial Registration:** Chinese Clinical Trial Registry (ChiCTR) ID: ChiCTR2300070320.

## 1 Introduction

Preterm infants, defined as newborns born before 37 completed weeks of gestation, remain a major global health concern. According to data from the World Health Organization, approximately 15 million preterm infants are born worldwide each year, with China contributing 1.17 million cases and ranking third globally (1). Concurrently, adjustments to China's reproductive policies have increased the proportion of pregnancies among women of advanced maternal age and other high-risk pregnancies, contributing to rising rates of preterm birth (2–4). A critical challenge faced by these infants is impaired suck-swallow-breath (SSwB) coordination, particularly among those born before 34 weeks of gestation. This impairment compromises nutrient intake and contributes to feeding difficulties, growth delays, and long-term neurodevelopmental impairments (5–9). A systematic review has demonstrated that early nutritional intake is associated with postnatal neurodevelopment in preterm infants, highlighting the importance of early interventions to optimize developmental outcomes (10).

To address feeding difficulties and support neurodevelopment in preterm infants, oral motor intervention (OMI) has emerged as an effective early therapeutic strategy, demonstrating improvements in feeding performance, oral feeding readiness, and the efficiency of transition to independent oral feeding (11–13). OMI involves gentle stimulation of perioral and intraoral structures, including the cheeks, lips, jaw, tongue, soft palate, gums, and pharynx, to enhance oral muscle strength and sucking ability (14). The intervention typically consists of two components: oral stimulation (OS) and non-nutritive sucking (NNS). OS involves gentle stimulation of the perioral and intraoral regions to facilitate coordinated muscle activity involved in sucking and swallowing, while NNS involves providing a pacifier during tube feeding to support sucking development (15). Together, these components promote the coordination of the oral and pharyngeal muscles required for effective feeding (16, 17).

Among the available intervention protocols, the Premature Infant Oral Motor Intervention (PIOMI), developed by Lessen in 2011, was specifically designed for very preterm infants born at 26–29 weeks of gestation (18). Earlier programs include the Beckman Oral Motor Intervention (BOMI), which was developed for a broader population ranging from full-term newborns to adults (19), and Fucile's Oral Motor Intervention Program (OMIs), which requires a 15-minute intervention session (20). In contrast, PIOMI provides a shorter and more standardized protocol, consisting of three minutes of oral stimulation and two minutes of non-nutritive sucking, administered once daily for seven consecutive days. This shorter intervention duration helps reduce the risk of fatigue in fragile preterm infants while maintaining therapeutic effectiveness, making PIOMI particularly suitable for clinical use in preterm infants.

Despite PIOMI being implemented in more than 20 countries (21), a standardized cross-cultural adaptation for mainland China has not yet been established. Although Chinese nurses recognize the importance of oral motor interventions, complex procedural requirements and longer intervention duration limit their routine use in clinical practice (22). Therefore, a brief and standardized protocol adapted to the Chinese healthcare context is needed to facilitate implementation and ensure intervention fidelity.

This study aimed to translate and cross-culturally adapt the Premature Infant Oral Motor Intervention (PIOMI) into a mainland Chinese version (CMV-PIOMI), evaluate the content validity and implementation consistency of the adapted protocol among neonatal nurses, and conduct a pilot randomized controlled trial to examine its clinical feasibility and preliminary effects on oral sucking performance in very preterm infants.

## 2 Methods

This study was conducted in two phases: (1) cross-cultural translation and adaptation of the Premature Infant Oral Motor Intervention (PIOMI) protocol, and (2) a parallel-group pilot randomized controlled trial to evaluate the feasibility and preliminary effects of the CMV-PIOMI.

### 2.1 Phase 1: translation, cross-cultural adaptation, and assessment of content validity and implementation consistency

#### 2.1.1 Instrument description

The Premature Infant Oral Motor Intervention (PIOMI) was operationalized as a procedural checklist for this study. Each step is performed using standardized techniques with specified durations, such as cheek C-stretch (30 s per cheek) and lip roll (30 s for each lip). The entire intervention requires approximately five minutes, including three minutes of oral stimulation and two minutes of non-nutritive sucking.

To support consistent implementation, Dr. Lessen developed an operational checklist that evaluates each step using a four-point scale (0 = no attempt, 1 = partially completed, 2 = completed with errors, and 3 = completed exactly as described). In addition, the correctness of the step sequence (order) and the accuracy of timing are each evaluated using a four-point scale. Therefore, the checklist includes ten scoring items (eight technique items, one order item, and one time item), yielding a total score ranging from 0 to 30.

The checklist demonstrated high reliability in the original study, with interrater reliability of 97.57%, interuser reliability of 97.59%, and test–retest reliability of 97.58% (23). In this study, these scores were used to assess the consistency of intervention implementation rather than psychometric properties.

#### 2.1.2 Instrument translation and cultural adaptation

The translation process followed Brislin's cross-cultural research methods (24) and established guidelines for translation and adaptation (25). This approach provides a systematic framework for cross-cultural adaptation, ensuring linguistic equivalence and cultural appropriateness. The six-step process (forward translation, synthesis, back-translation, expert review, pretesting, and assessment of implementation consistency) has been widely used in healthcare research, including the cross-cultural adaptation of the Neonatal/Infant Braden Q Scale (26) and the Turkish adaptation of PIOMI (21). Given the procedural nature of PIOMI and the need to maintain intervention fidelity across cultural contexts, this method provides a rigorous and reproducible approach for adapting the protocol. The translation process consisted of the following six steps.

**Preparation**: First, we sent an email to the original author of the development of PIOMI and obtained her consent to cross-culturally adapt PIOMI into a mainland Chinese version. We then established a cross-cultural adaptation research team.

##### 2.1.2.1 Step 1: forward translation

The original PIOMI was translated into Simplified Chinese by two independent translators. Both translators have lived in mainland China for an extended period and possess a strong understanding of mainland Chinese culture. Both have professional proficiency in English. One translator had extensive experience in neonatal care and was familiar with medical terminology. The other translator had a medical background but no neonatal care experience. The two translators independently translated the PIOMI into translated version 1 (TL1) and translated version 2 (TL2).

##### 2.1.2.2 Step 2: synthesis Ⅰ: comparison of the TL1 and TL2

A third translator with study experience in English-speaking countries compared the differences between TL1 and TL2 in terms of sentence meaning, word meaning, and context. After discussion, the three translators reached a consensus, and the preliminary initial translated version of the PIOMI (PI-TL) was created.

##### 2.1.2.3 Step 3: back-translation of PI-TL

Two other translators were involved in this step to generate two back-translated versions. The first was an English language professional with a medical background but was unaware of the original version of the PIOMI. The second translator was Chinese, who lived in America for a long time and was familiar with daily language and basic healthcare terminology but had no knowledge of PIOMI. This process produced two back-translated versions, B-TL1 and B-TL2.

##### 2.1.2.4 Step 4: synthesisⅡ: comparison of B-TL1, B-TL2 and source language PIOMI

A native Chinese-speaking medical doctor with advanced English proficiency compared B-TL1 and B-TL2 with the original PIOMI and resolved any discrepancies. This process was repeated until the back-translated version closely matched the original PIOMI. A pre-final translated Chinese version of the PIOMI (P-FTL) was generated.

##### 2.1.2.5 Step 5: pretest (cross-cultural adaptation using e-delphi)

Cross-cultural adaptation aims to ensure equivalence between the source and translated versions while maintaining content validity across different cultural contexts (26). In this study, the pretesting phase included two components: expert evaluation using the Delphi method and confirmation with the original author.

e-Delphi Process: The e-Delphi method, an online form of the classical Delphi technique, was used to assess the clarity, feasibility, and cultural appropriateness of the pre-final Chinese version of the PIOMI (27, 28).

Expert Selection: Experts were recruited based on the following criteria: (1) senior nursing managers or frontline clinical nurses with senior professional titles; (2) at least five years of experience in neonatal care; (3) specialization in neonatal nursing; (4) willingness to participate and provide expert opinions. A total of 21 experts were invited. Previous methodological studies indicate that a panel of 10–30 experts is generally considered adequate for Delphi studies (29).

Evaluation Criteria: Experts were asked to evaluate three aspects of each item in the Chinese version of the PIOMI using a 5-point Likert scale: (1) clarity of linguistic presentation, (2) feasibility of implementation in mainland China, and (3) cultural appropriateness (1 = strongly disagree, 2 = relatively disagree, 3 = agree, 4 = relatively agree, 5 = strongly agree). Each item was followed by an open-ended question to collect experts' opinions and suggestions for improvement.

Consensus Procedure: The e-Delphi method was conducted for two rounds. After the first round, researchers analyzed experts' comments and revised the items accordingly. The second round was conducted with the same experts until consensus was reached.

Confirmation with Original Author: Following the Delphi consensus, the research team created a video demonstration of the preliminary Chinese version of PIOMI. The video and revised Chinese version were sent to the original author via email, followed by an online meeting through Zoom software. The original author evaluated the content and provided guidance on operational details to refine the Chinese mainland version of PIOMI (CMV-PIOMI).

##### 2.1.2.6 Step 6: assessment of content validity and implementation consistency

Content Validity Assessment: Content validity was evaluated using the item-level content validity index (I-CVI) and the scale-level content validity index (S-CVI/Ave). During the second round of the Delphi consultation, experts were asked to rate the relevance of each item of the P-FTL PIOMI to oral motor intervention using a 4-point Likert scale (1 = not relevant, 2 = somewhat relevant but requires major revision, 3 = quite relevant but requires minor revision, and 4 = highly relevant). The I-CVI was calculated as the proportion of experts rating an item as 3 or 4. The S-CVI/Ave was calculated as the average of the I-CVI values across all items. An I-CVI value greater than 0.78 and an S-CVI/Ave value greater than 0.90 were considered indicative of acceptable content validity.

Implementation Consistency Assessment: In this study, PIOMI procedures performed by neonatal nurses were independently evaluated by two trained researchers using the PIOMI checklist. Both researchers had received formal PIOMI training and certification from Professor Lessen and were able to accurately perform and assess the intervention. The checklist evaluates ten items (eight technique items, one order item, and one time item), yielding a total score ranging from 0 to 30. The average score of the two raters was used as the final score.

All participating nurses had no prior exposure to PIOMI. To examine the consistency of implementation, the procedures were evaluated twice with a two-week interval to minimize potential recall bias. The scores from the two assessments were compared using a paired samples *t*-test. A *p*-value greater than 0.05 indicated that no statistically significant difference between the two operational scores, suggesting stable and consistent implementation of the CMV-PIOMI protocol.

**Figure 1:**
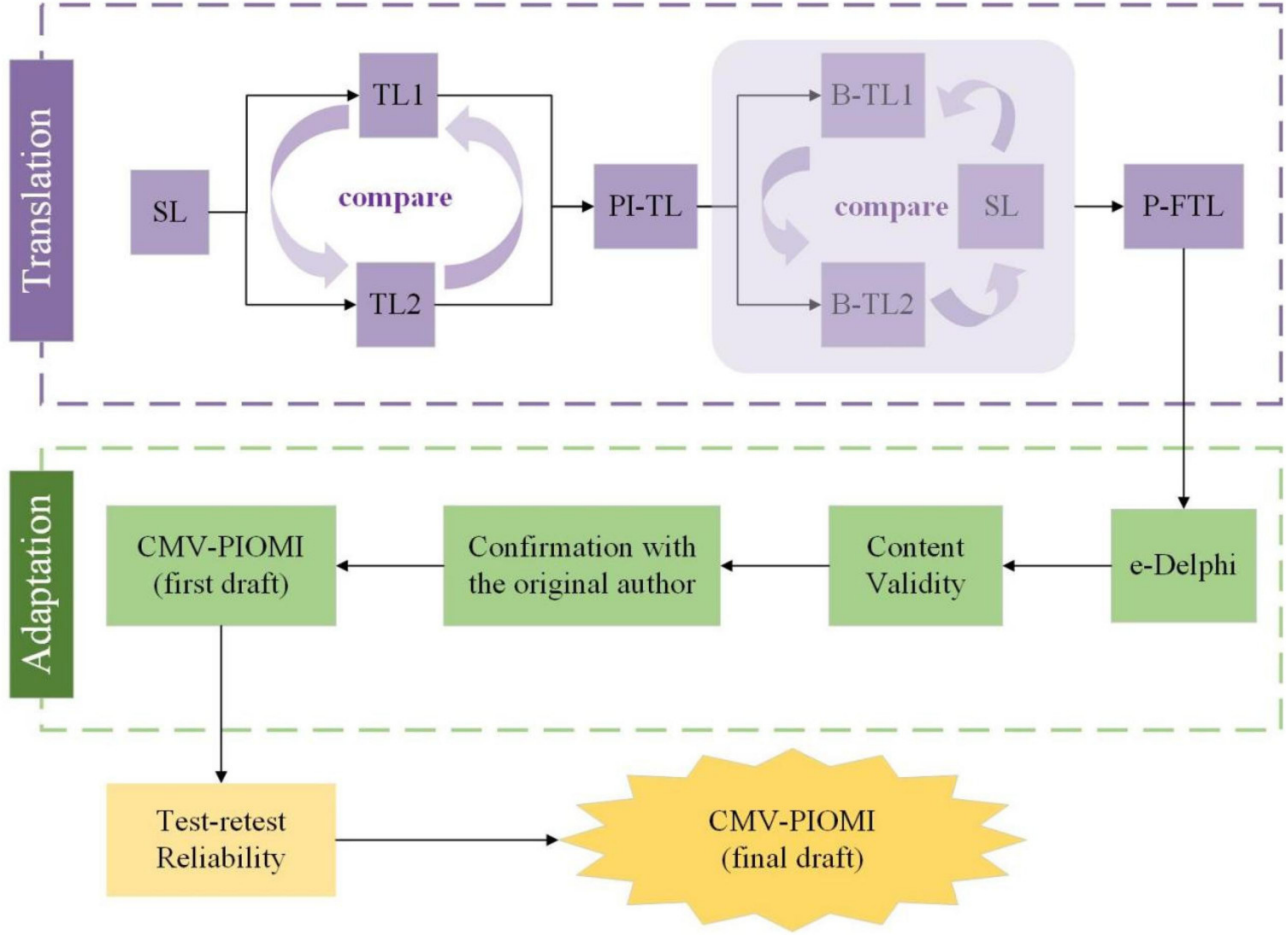
The Steps for the cross-cultural translation and adaptation of PIOMI into Chinese mainland version.

The overall process of cross-cultural translation and adaptation of PIOMI into the Chinese mainland version (CMV-PIOMI) is illustrated in Figure 1.

### 2.2 Phase 2: parallel-group pilot randomized controlled trial

#### 2.2.1 Study setting and sampling

The study was conducted at a large tertiary-level Class A maternal and child health specialty hospital in Chengdu. The subjects in the application stage were very preterm infants admitted to the neonatal intensive care unit (NICU).

Sample Size Estimation: The required sample size was calculated using G*Power software. Assuming a medium effect size (W = 0.5), a significance level of *α* = 0.01, statistical power of 1−*β* = 0.90, and degrees of freedom (df) = 1, the minimum sample size was estimated to be 60 participants. Considering a potential dropout rate of 20%, the minimum sample size was increased to 75. To ensure equal allocation between groups, the final sample size was set at 76 cases.

Randomization and Blinding: Participants were randomly assigned to the intervention or control group using a computer-generated randomization sequence. The allocation sequence was concealed using 76 sequentially numbered, opaque, sealed envelopes. Each envelope contained a group assignment corresponding to the randomization code.

After obtaining informed consent, participants were assigned to groups by a research assistant (RA) according to their enrollment order and the code contained in the envelope. Participants, data collectors, and statisticians were blinded to group allocation. Due to the nature of the intervention, the nurses delivering the CMV-PIOMI intervention could not be blinded.

#### 2.2.2 Data collection

Standard Care Protocol: The control group received standard treatment and care, which included incubator temperature regulation, respiratory support as needed, gavage feeding according to unit protocol, developmental supportive care, and routine nursing assessments. The intervention group received the same standard treatment and care as the control group, in addition to the Chinese adaptation of the CMV-PIOMI.

CMV-PIOMI Implementation: The CMV-PIOMI was delivered by research nurses who received specialized training and certification from Dr. Lessen. Each infant in the intervention group was assigned to one primary nurse for the duration of the 7-day intervention. The CMV-PIOMI was administered once daily for seven consecutive days, with each session lasting approximately five minutes (three minutes of oral stimulation and two minutes of non-nutritive sucking).

Outcome Measurement: The Neonatal Oral Motor Assessment Scale (NOMAS) was used to evaluate the sucking ability of extremely preterm infants, encompassing core parameters of neonatal oral motor function, including sucking initiation, intensity, and rhythm. This scale was established by Braun et al. in 1985 and was subsequently revised by Palmer et al. The scale categorizes the newborn's sucking status into three types: normal sucking, sucking disorder, and sucking dysfunction. The scale has demonstrated good reliability and validity, with Cohen's kappa coefficients ranging from 0.67 to 0.80, Cronbach's *α* coefficient of 0.75–0.76, and test–retest reliability of 0.89 (30).

### 2.3 Statistical data analysis

Data were entered into Excel and analyzed using SPSS version 26.0. Descriptive analyses were used to describe the general information of participants, including frequency, ratios, means, and standard deviations. For Phase 1 (implementation consistency assessment), Pearson correlation analysis and paired samples *t*-tests were used to compare the scores of the two assessments. A *P* value < 0.05 was considered statistically significant. For Phase 2 (clinical application phase), continuous variables were first assessed for normality using skewness statistics, histograms with normal curves, and Q-Q plots. Normally distributed data were presented as mean ± SD, whereas non-normally distributed data were expressed as median and interquartile range (IQR). Categorical variables were reported as frequency and percentage. The chi-square test, independent samples *t*-test, and Mann–Whitney *U*-test were used to compare between-group differences. Effect sizes were reported as risk difference (RD) with 95% confidence interval (CI) for categorical variables, and mean difference (MD) with 95% CI for continuous variables.

### 2.4 Ethical considerations

This study was prospectively registered in the Chinese Clinical Trial Registry (ChiCTR2300070320) and approved by the Ethics Committee of Chengdu Women's and Children's Central Hospital (approval number: Scientific Research Ethics Review 2023 (23)-2). Written informed consent was obtained from the parents or legal guardians of all preterm infants prior to enrollment.

## 3 Results

### 3.1 Phase 1 results: cross-cultural adaptation, content validity, and implementation consistency

#### 3.1.1 e-Delphi process

A total of 21 experts were invited to participate in the cross-cultural adaptation of the Chinese mainland version of PIOMI. Demographic characteristics of the experts are provided in Table 1. The e-Delphi consultation was conducted between February and May 2022. Members of the research team contacted the experts in advance to obtain their informed consent, and questionnaires were distributed and collected via WeChat or email, with experts asked to complete each round within 1–2 weeks.

**Table 1:** Demographic characteristics of expert panel (*n* = 21).

No.	Age (years)	Working experience (years)	Professional title	Educational background	Positions	Research/Job Direction
1	44	20	Chief Nurse Practitioner	PhD	Deputy Director of Nursing	Neonatal care
2	31	10	nurse practitioner in charge	Undergraduate	Clinical Nurse	Breast feeding
3	43	19	nurse practitioner in charge	Master's Degree	Head Nurse	Care of premature babies
4	52	35	Chief Nurse Practitioner	Undergraduate	Clinical Nurse	Developmental care techniques for premature babies
5	56	36	Chief Nurse Practitioner	Undergraduate	Head Nurse	Neonatal care
6	38	15	nurse practitioner in charge	Undergraduate	Clinical Nurse	Care of premature babies
7	47	25	Deputy Chief Nurse Practitioner	Undergraduate	Head Nurse	Critical neonatal care
8	40	25	Chief Nurse Practitioner	Undergraduate	Clinical Nurse	Clinical Nursing Management & Teaching
9	35	14	Chief Nurse Practitioner	Master's Degree	Clinical Nurse	Care of extremely preterm infants
10	42	23	Deputy Chief Nurse Practitioner	Master's Degree	Head Nurse	Neonatal care and management
11	42	18	Deputy Chief Nurse Practitioner	Undergraduate	Head Nurse	Care of premature babies
12	50	30	Chief Nurse Practitioner	Undergraduate	Head Nurse	Neonatal critical care
13	55	35	Deputy Chief Nurse Practitioner	Undergraduate	Section Nurse Manager	Neonatal critical care
14	52	32	Deputy Chief Nurse Practitioner	Undergraduate	Clinical Nurse	Nutrition for preterm babies & Developmental care techniques for premature babies
15	38	12	Chief Nurse Practitioner	Master's Degree	Head Nurse	Paediatric nursing
16	34	12	Chief Nurse Practitioner	Undergraduate	Clinical Nurse	Neonatal intensive care
17	40	22	Chief Nurse Practitioner	Undergraduate	Assistant head nurse	Nursing clinical quality control and management
18	46	26	Deputy Chief Nurse Practitioner	Undergraduate	Head nurse	Rehabilitation nursing
19	29	6	Junior Therapist	Undergraduate	Rehabilitation therapist	Pediatric Speech and Swallowing Therapy
20	40	20	Senior Therapist	Undergraduate	Chief therapist	Pediatric Speech and Swallowing Therapy
21	40	21	Chief Nurse Practitioner	Undergraduate	Associate head nurse	Family-centered care

In Round 1, all 21 experts responded. A total of 38 amendments were proposed, primarily focused on linguistic expression and cultural appropriateness. The research team discussed and revised the items according to expert opinions.

In Round 2, eighteen experts responded (response rate: 85.7%). Six amendments were proposed, focusing on operational refinements and clarification of procedural details. The research team analyzed the comments and made final revisions until consensus was achieved.

Consensus was reached for all items after two rounds of consultation.

#### 3.1.2 Content validity

During the second round of the e-Delphi process, 18 experts responded. These 18 experts were asked to rate the relevance of each item of the CMV-PIOMI to oral motor interventions using the 4-point Likert scale. The item-level content validity index (I-CVI) ranged from 0.88 to 1.00, and the scale-level content validity index (S-CVI/Ave) was 0.96, indicating acceptable content validity.

#### 3.1.3 Implementation consistency

A total of 117 NICU nurses participated in the implementation consistency assessment. The majority were female (*n* = 114, 97.44%). The mean age was 29.32 ± 3.91 years, and the mean clinical working experience was 6.89 ± 4.05 years. Detailed participant characteristics are presented in Table 2.

**Table 2:** Characteristics of nurses participating in the implementation consistency assessment (*n* = 117).

Variable	*n* (%)/Mean ± SD
Age (years)	29.32 ± 3.91
Working experience (years)	6.89 ± 4.05
NICU working experience	
0–5 years	59 (50.43%)
6–10 years	34 (29.06%)
11–15 years	20 (17.09%)
＞15 years	4 (3.42%)
Gender	
Female	114 (97.44%)
Male	3 (2.56%)
Professional title	
Junior	84 (71.79%)
Intermediate	32 (27.35%)
Senior	1 (0.85%)
Educational background	
Junior college	45 (38.46%)
Undergraduate	72 (61.54%)

Data are presented as mean ± SD for continuous variables and *n* (%) for categorical variables. NICU, neonatal intensive care unit.

None of the participating nurses had prior exposure to PIOMI. Each nurse performed the CMV-PIOMI procedure on preterm infant models twice, with a two-week interval between assessments. The mean operational score was 27.95 ± 0.48 in the first assessment and 27.99 ± 0.45 in the second assessment. Pearson correlation analysis showed a significant correlation between the two assessments (*r* = 0.683, *p* < 0.001). The paired-samples *t*-test revealed no statistically significant difference between the two operational scores (*t* = 1.117, *p* = 0.266), indicating that the CMV-PIOMI protocol could be implemented consistently by trained nurses.

#### 3.1.4 Final version of CMV-PIOMI

**Figure 2:**
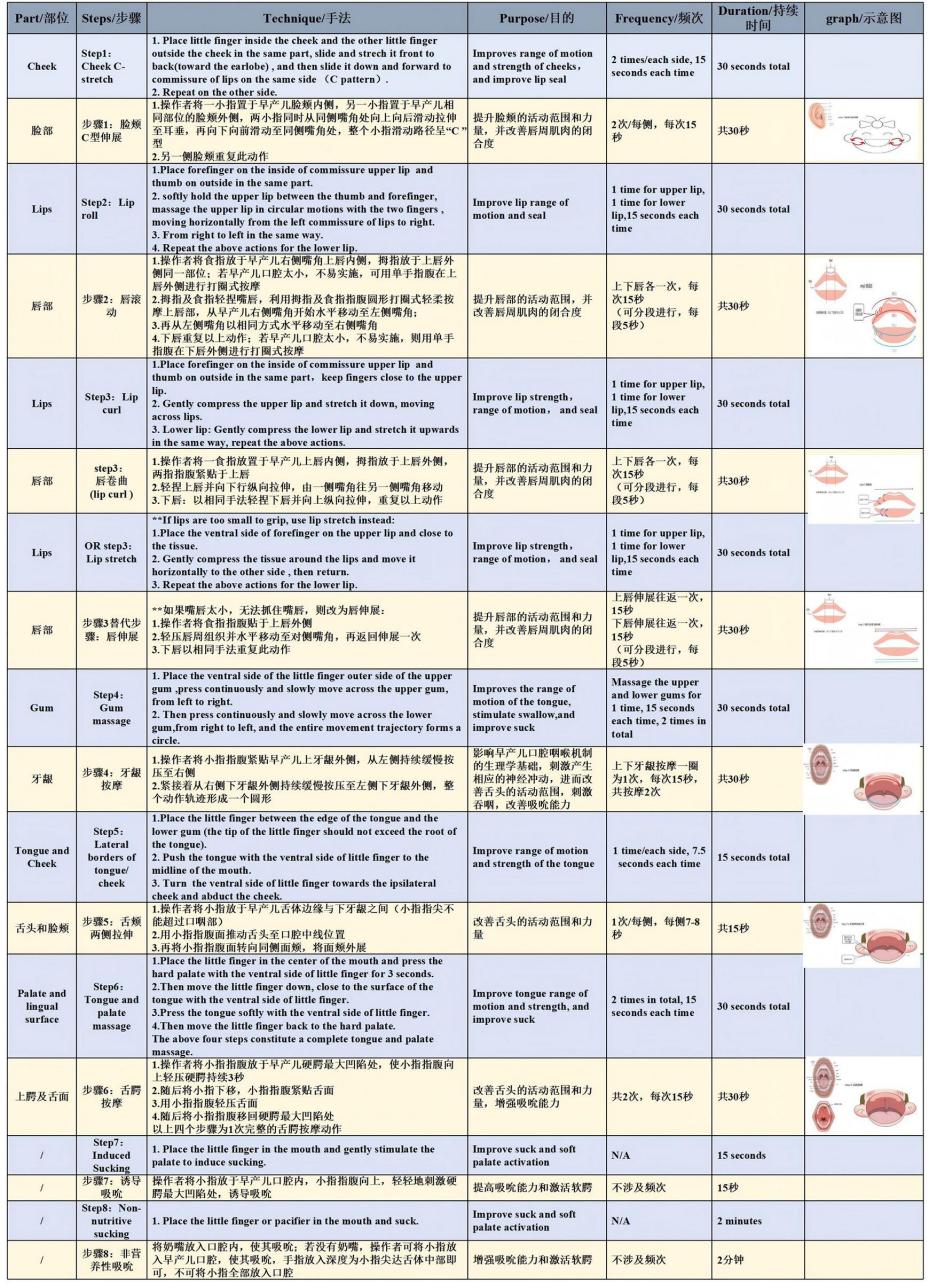
Operational diagram of the mainland Chinese version of the premature infant oral motor intervention (CMV-PIOMI).

The final Chinese mainland version of PIOMI(CMV-PIOMI) contains eight steps: cheek C-stretch [2 items];lip roll [4 items];lip curl [3 items];lip stretch [3 items];gum massage [2 items];lateral borders of tongue/ cheek [3 items];midblade of tongue/palate [4 items];elicit a suck [1 item];support for non-nutritive sucking [1 item]. Researchers had linguistically adapted the original English version of PIOMI to make the linguistic expression of PIOMI more in line with the expression habits of mainland China. Figure 2 shows the detailed operating steps.

### 3.2 Phase 2 results: pilot randomized controlled trial

#### 3.2.1 Baseline characteristics

The baseline characteristics of the intervention and control groups are presented in Table 3. No statistically significant differences were observed between the two groups with respect to sex, birth weight, birth length, head circumference, age, or Apgar scores (all *p* > 0.05), indicating that the two groups were comparable at baseline.

**Table 3:** Baseline characteristics of the two groups of very preterm infants (*n* = 76).

Items	Intervention group	Control group	Statistic	*P* value
Sex, *n* (%)			1.362	0.243
Male	25 (65.8%)	20 (52.6%)		
Female	13 (34.2%)	18 (47.4%)		
Sucking condition			0.370	0.543
Normal sucking	2 (5.3%)	1 (2.6%)		
Abnormal sucking	36 (94.7)	37 (97.4%)		
Birth weight (g)	1,311.84 ± 229.957	1,351.84 ± 243.697	0.064	0.800
Birth length (cm)	38.13 ± 2.327	38.53 ± 2.480	0.276	0.601
Birth head circumference (cm)	27.64 ± 1.408	27.91 ± 2.247	1.687	0.198
Age in days	7 (4, 11)	7 (5, 10)	−0.109	0.913
Apgar score at 1 min	8 (8, 9)	8 (8, 9)	−0.227	0.821
Apgar score at 5 min	9 (9, 9)	9 (9, 9)	−0.472	0.637
Apgar score at 10 min	9 (9, 9)	9 (9, 9)	−0.103	0.989

Data are presented as *n* (%), mean ± SD, or median (IQR). IQR, interquartile range. *χ*^2^, chi-square test; *t*, independent samples *t*-test; Z, Mann–Whitney *U-*test statistic.

#### 3.2.2 Intervention outcomes

At baseline, normal sucking patterns were observed in 5.3% (2/38) of the intervention group and 2.6% (1/38) of the control group. After the 7-day intervention, the proportion of infants with normal sucking patterns was significantly higher in the intervention group (50.0%, 19/38) compared to the control group (21.1%, 8/38) (*χ*^2^ = 6.951, *p* = 0.008, 95% CI: 7.0%–47.2%). The comparison of outcomes between the two groups after the intervention is presented in Table 4.

**Table 4:** Comparison of outcomes between two groups after intervention (*n* = 76).

Outcome	Intervention group (*n* = 38)	Control group (*n* = 38)	Statistic	*P* value	95% CI
Sucking condition			6.951	0.008	0.07–0.47
Normal sucking	19 (50.0%)	8 (21.1%)			
Abnormal sucking	19 (50.0%)	30 (78.9%)			
Weight (g)	1,560.00 ± 232.59	1,623.68 ± 293.57	0.222	0.639	−184.75–57.38
Length (cm)	40.02 ± 2.40	40.04 ± 2.03	1.439	0.234	−1.03–1.00
Head circumference (cm)	28.74 ± 1.28	28.37 ± 1.69	0.375	0.542	−0.32–1.05

Data are presented as mean ± SD or *n* (%). The chi-square test was used for categorical variables, and the independent samples *t*-test was used for continuous variables. The statistic column represents the test statistic (*χ*^2^ or *t*). 95% CI = 95% confidence interval for the difference between groups (risk difference for sucking condition, mean difference for continuous variables). CI, confidence interval.

## 4 Discussion

The aim of this study was to translate and cross-culturally adapt the Premature Infant Oral Motor Intervention (PIOMI) for use in mainland China, and to preliminarily evaluate its feasibility and potential effects in a pilot randomized controlled trial. Oral motor interventions have demonstrated promising results in improving feeding outcomes in preterm infants. Previous studies utilizing Fucile's 15-minute protocol have shown an accelerated transition to oral feeding (31). However, the longer duration may present practical challenges in clinical settings and may increase the risk of fatigue in preterm infants. To address these concerns, Professor Lessen developed the PIOMI protocol specifically designed for very preterm infants, with a shortened 5-minute intervention that enhances feasibility in routine clinical practice. Meta-analytic evidence supports the efficacy of PIOMI, indicating that it may improve feeding progression and reduce hospitalization duration in preterm infants (32). However, further validation across diverse clinical settings is necessary to confirm its broader applicability and effectiveness.

The cross-cultural adaptation of PIOMI was conducted following established methodological frameworks, including Brislin's cross-cultural research method and widely recognized guidelines for translation and adaptation (24, 25). Throughout the process, we maintained communication with the original author to ensure that the adapted version remained faithful to the original protocol. The two-round e-Delphi process resulted in several linguistic and cultural adjustments aimed at enhancing the clarity and clinical applicability of the Chinese version of PIOMI in the local healthcare context.

Content validity and implementation consistency are essential considerations when adapting intervention protocols for different clinical settings. Content validity ensure that the intervention components are appropriate and relevant for the target population (33). Implementation consistency reflects whether the intervention can be delivered reliably and consistently across different occasions (34), which is essential for ensuring its real-world applicability. In the present study, implementation consistency was assessed using the PIOMI operational checklist, which evaluates adherence to the procedural steps of the intervention rather than psychometric properties of a measurement instrument. The results indicated acceptable consistency among trained nurses, suggesting that the adapted protocol can be implemented with reasonable fidelity.

The pilot randomized controlled trial provided preliminary data on the potential effects of CMV-PIOMI in very preterm infants. Oral motor interventions have been reported to facilitate the transition from tube feeding to oral feeding and improve feeding outcomes in preterm infants (35–37). Previous studies applying the PIOMI protocol have also reported shorter time to achieve independent oral feeding and full oral feeding schedules (36). In this study, the intervention group demonstrated a significantly higher proportion of infants with normal sucking patterns compared to the control group after the 7-day intervention. These findings align with previous reports suggesting oral motor interventions may support the development of feeding skills in preterm infants. However, the results should be interpreted cautiously due to the small sample size and single-center design. The 95% confidence intervals for the risk difference (7.0%–47.2%) indicate a level of uncertainty regarding the magnitude of the observed effect.

## 5 Limitations and strength

This study has several methodological limitations that should be acknowledged. First, the cross-cultural adaptation and pilot trial were conducted at a single tertiary hospital, which may limit the generalizability of the findings to other settings with different resources and patient populations. Second, the expert panel primarily consisted of neonatal nursing specialists, and although all had extensive experience in neonatal feeding, the absence of speech-language specialists may be considered a limitation, given the oral-motor nature of the intervention. Third, the relatively small sample size in the pilot RCT resulted in wide confidence intervals, limiting the ability to draw definitive conclusions about the intervention's effectiveness. Larger, multicenter studies are needed to further evaluate the effectiveness and generalizability of the CMV-PIOMI protocol.

Despite these limitations, this study has several strengths. First, the PIOMI protocol was systematically translated and cross-culturally adapted for use in mainland China, improving its relevance and applicability in the local clinical context. Second, the adaptation process followed established methodological frameworks, including Brislin's translation model and the e-Delphi expert consultation method, which ensured transparency and methodological rigor. Third, the development of a culturally adapted operational guide and nurse training procedure supports the practical implementation of CMV-PIOMI in neonatal nursing practice.

## 6 Conclusion

The Chinese mainland version of PIOMI demonstrated acceptable content validity and implementation consistency following cross-cultural adaptation. The pilot randomized controlled trial provided preliminary evidence suggesting potential benefits for oral sucking patterns in very preterm infants, though these findings require confirmation in larger, adequately powered studies. The CMV-PIOMI protocol offers a potentially feasible approach for oral motor intervention in Chinese neonatal care settings, pending further validation.

## Data Availability

The original contributions presented in the study are included in the article/Supplementary Material, further inquiries can be directed to the corresponding author.
